# Abstracts of the 3rd Annual International Motor City Diabetes Symposium

**DOI:** 10.1155/EDR.2001.153

**Published:** 2001

**Authors:** Donald F. Stenier

**Affiliations:** University of Chicago Chicago IL USA


*Int. Jnl. Experimental Diab. Res.*, Vol. 2, pp. 153–165
Reprints available directly from the publisher
Photocopying permitted by license only

© 2001 OPA (Overseas Publishers Association) N.V.
Published by license under
the Harwood Academic Publishers imprint,
part of Gordon and Breach Publishing,
a member of the Taylor & Francis Group.
Printed in the U.S.A.

### Abstracts of the 3rd Annual International Motor City Diabetes Symposium*

DETROIT, MICHIGAN, OCTOBER 20–21, 2000

### The Proinsulin C-peptide Discovery, Structural Aspects and Role(s)

DONALD F. STENIER

*University of Chicago, Chicago, IL*

The C-peptide, which consists of the connecting segment of proinsulin minus the basic resides at each end, is a product of proinsulin processing that is stored and cosecreted with insulin in essentially equimolar amounts from the beta cell. It has been isolated from the pancreas of man and a number of other species and studies of its secretion and metabolic clearance have established that it is a valuable and reliable indicator of beta-cell secretory function in normal and diabetic subjects. Unlike insulin, which is rapidly removed from the circulation by receptor binding, the C-peptide has a prolonged half-life without significant tissue sequestration. In mammals the C-peptide ranges in length from 26-31 residues, has a 15-fold greater rate of mutation acceptance than insulin and is cleaved internally prior to secretion in some species, *i.e.*, the dog, fox and others. It has a somewhat restricted composition, notable for the absence of aromatic residues, and usually contains four or five acidic residues, which offset the four basic amino acids at the cleavage sites thus maintaining a very similar isoelectric point in proinsulin to that of insulin. The foremost biosynthetic role of the C-peptide clearly is to serve as a flexible linker between the insulin B and A chains so as to facilitate the proper folding of the peptide chain and disulfide bond formation. The C-peptide does this mainly by converting the bimolecular reaction of chain combination to a more efficient monomolecular reaction, but this function can also be accomplished by much shorter or even totally deleted connecting segments. The strong conservation of the length of the C-peptide in evolution, however, is consistent with the requirement for its efficient excision from proinsulin during its conversion to insulin; experiments with truncated C-peptides show marked reductions in conversion efficiency. Optimal binding of proinsulin to the prohormone convertases for cleavage imposes significant constraints on the length and properties of the C-peptide, as will be discussed. Moreover, the C-peptide may play a role in sorting and must be compatible with other constituents of the secretory granule in which it is stored, *e.g.*, insulin, islet amyloid peptide and others. In addition to these constraints, the mammalian C-peptides may have acquired some additional beneficial functions that complement insulin action.

*Theme: "C-peptide: Has its time come? From mechanism of action to its use in diabetes".

### Overview of C-peptide Developments During the Last 10 Years

JOHN WAHREN

*Section of Clinical Physiology, Department of Surgical Sciences, Karolinska Institute, SE 171 77 Stockholm, Sweden*

The observation in 1992 that C-peptide in physiological concentrations can correct glomerular hyperfiltration and increase renal blood flow in patients with type 1 diabetes [1] became the starting point for continued studies of C-peptide physiology in Stockholm These findings sub-sequently led to more detailed studies of renal function in diabetes, evaluation of C-peptide-blood flow interactions, studies of C-peptide's cellular interactions and signal transduction, and investigations of C-peptide and nerve function in diabetes.

The initially observed C-peptide effect on renal function has been confirmed and extended in both experimental studies in streptocotozin-diabetic (STZ) rats and type 1 diabetes patients. Thus, C-peptide replacement for periods of two weeks to three months in animals and patients with early nephropathy results in diminished glomerular hyperfiltration and reduced urinary albumin excretion. [2–4] The effect of C-peptide on blood flow has been further examined using the forearm model in type 1 diabetes patients. A significant increase in forearm blood flow is observed during *i.v.*, infusion of C-peptide in the resting state.[5] Likewise, infusion of C-peptide during rhythmic forearm exercise results in a marked increase in both blood flow and oxygen uptake. [6] The latter finding prompted an evaluation of C-peptide's possible influence on $Na^+$, $K^+$-ATPase. It could be shown that C-peptide has the capacity to stimulate renal tubular cell $Na^+$, $K^+$-ATPase activity in a concentration-dependent manner, probably by activating a receptor coupled to a pertussis-toxin sensitive G-protein with subsequent activation of $Ca^{2+}$-dependent intracellular signaling pathways. [7] It could also be demonstrated in endothelial cells that the C-peptide-induced rise in intracellular $Ca^{2+}$ stimulates endothelial nitric oxide synthase activity and causes release of NO, [8] thereby helping to explain the effects of C-peptide on blood flow.

It has been suggested that C-peptide effects may be mediated by non-chiral cell membrane interactions similar to those of antimicrobial peptides. [9] However, there is now new evidence that C-peptide binds to specific cell surface receptors which has been obtained using fluorescence correlation spectroscopy. Specific binding of human C-peptide could be demonstrated for human tubular cells, skin fibroblasts and saphenous venous endothelial cell. [10] $K_{ass}$ for C-peptide binding was $\sim 3\cdot10^{-9}\ M^{-1}$. Insulin, IGF I and II, proinsulin and NPY showed no cross-reactivity. The C-peptide binding isotherm indicates that half saturation is reached at approximately 0.9 nM. This finding explains the consistent observation that no effects of C-peptide can be observed in healthy individuals or animals, in whom the ambient C-peptide concentration will be sufficient to cause saturation of C-peptide receptors.

Reduced levels of endoneurial $Na^+$, $K^+$-ATPase and diminished blood flow have been reported in diabetes. The effect of C-peptide on motor nerve conduction velocity[9] was observed in STZ-treated rats. In addition, a significant improvement of autonomic nerve function, as evidenced by increased heart rate variability during breathing, has been demonstrated in patients with type 1 diabetes and signs of autonomic neuropathy. [11]

In conclusion, the available data establish that C-peptide is not as biologically inert as previously believed. Instead, it now emerges as an active peptide hormone with potentially important physiological effects. The possibility should be considered that C-peptide replacement together with insulin administration might be beneficial in type 1 diabetes patients.

### *References*

[1] Sjöberg, S. *et al.* (1991). Diabetologia, **34,** 423–428.
[2] Johansson, B.-L. *et al.* (1993). *J. of Clinical Endoc. Metab.*, **77,** 976–981.
[3] Sjöquist, M. *et al.* (1998). *Kidney International*, **54,** 758–764.
[4] Johansson, B.-L. *et al.* (2000). *Diabetic Medicine*, **17,** 181–189.
[5] Johansson, B.-L. *et al.* (1999). *Acta Physiol. Scand*, **165,** 39–44.
[6] Johansson, B.-L. *et al.* (1992). *Diabetologia*, **35,** 1151–1158.
[7] Ohtomo, Y. *et al.* (1996). *Diabetologia*, **39,** 199–205.
[8] Kunt, T. *et al.* (1998). *Diabetologia*, **41,** A176.
[9] Ido, Y. *et al.* (1997). *Science*, **277,** 563–566.
[10] Rigler, R. *et al.* (1999). *Proc. Natl. Aca. Sci.*, USA, **96,** 1318–1323.
[11] Johansson, B.-L. *et al.* (1996). *Diabetologia*, **39,** 687–695.

### Interaction and Dynamics of C-peptide and Cell Surface Bound Receptors

RUDOLF RIGLER[a], ALADDIN PRAMANIK[a], KARIN EKBERG[b], BO LENNART JOHANSSON[b], HANS JÖRNVALL[a] and JOHN WAHREN[b]

[a]*Departments of Biochemistry and Biophysics,* [b]*Clinical Physiology, Karolinska Institutet, S-17177 Stockholm, Sweden*

The development of single molecule detection and fluorescence correlation spectroscopy (FCS)[1] has provided the tools to analyze the interaction between fluorescently tagged ligands and their specific targets. We have been able to show that tetramethylrhodamin labelled C-peptide is able to bind specifically to receptors at the cell membrane. From, the study of the Brownian motion of C-peptide receptor complexes we were able to show[2,3] (i) a high affinity binding of the C-peptide to the receptor, (ii) a high specificity of the interaction which is related to a C-terminal pentapeptide sequence. Furthermore the data obtained from studies where the function of G-proteins was inhibited by pertussis toxin and a significant change of the binding affinity was found, suggest the existence of an allosteric network involving receptor conformations with different affinities to both C-peptide as well as G-protein.

Recent developments of 3 dimensional imaging of the C-peptide receptor complex in living cells and the simultaneous analysis of the Brownian mobility provides new insights in the functional activity of the C-peptide.

### *References*

[1] Eigen, M. and Rigler, R. (1994). *Proc. Natl. Acad. Sci.*, US, **91**, 5740.
[2] Rigler *et al.* (1999). *Proc. Natl. Acad. Sci.*, US, **96**, 1318.
[3] Wahren *et al.* (2000). *Am. J. Phys. Endocrin. Metab.*, **278**, E 759.

### Conservation and Variability: C-peptide *Versus* Other Biopeptides

HANS JÖRNVALL and ERIK NORDLING

*Department of Medical Biochemistry and Biophysics, Karolinska Institutet, SE-17177 Stockholm, Sweden*

Observations of clinical effects of C-peptide in patients with diabetes type I,[1] combined with measurable effects of C-peptide and its fragments on cellular $Na^{+}K^{+}$ ATPase activity,[2, 3] and of direct cellular binding interactions of C-peptide,[4] have triggered interest in C-peptide and its possible role as a biologically active hormone. Still, decisive break-throughs in definitions of receptor(s) have not been reported, and contradictory reports still exist regarding which segments of C-peptide that may be important, or through which mechanisms (*cf.*[5,6]). For these reasons, it is of interest to study the chemical variability of C-peptide as deduced from its molecular evolution and to compare that with the species variability of conventional peptide hormones. Notably, C-peptide is now known in PIR databank entries for no less than 37 vertebrate species, and insulin for 72 species. We have correlated the patterns for these peptides with those for vasoactive intestinal polypeptide (VIP, forms from 17 species), secretin (9 forms), glucagon (31 forms), insulin-like growth-factor 1 (IGF1, 23 forms), IGF2 (26 forms), gastrin (14 forms), gastrin releasing peptide (GRP, 10 forms), parathormone (15 forms), substance P (20 forms), cholecystokinin (CCK, 20 forms), somatostatin (28 forms), gastric inhibitory polypeptide (GIP, 5 forms) and pancreatic polypeptide (24 forms). Several observations are apparent from this set of 15 different bioactive peptides.

First of all, C-peptide is the peptide with the largest species variability. Its pattern is markedly different from those for both insulin and IGFs. Of course, this C-peptide variability is a concern in relation to a specific function of C-peptide, since conventional peptide hormones usually have some segment with species conservation, constituting an active site in receptor binding interactions. In short, observations on the now large set of species variants do not immediately define a biologically active segment for C-peptide.

Second, the conservation patterns of all the 15 biopeptides fall into four groups of different variability, with C-peptide in the most variable group, but within that group not that enormously more variable than its fellow peptides in the same group (GRP and parathormone). Hence, from an evolutionary point of view, based on observations of the species variability pattern, the case for C-peptide as a hormone is still, in spite of its variability, open for consideration. Of the 15 biopeptides in the four sub-groups, remaining peptides, in the order of decreasing variability, are a group of moderately variable peptides (CCK, secretin, pancreatic polypeptide and gastrin); a group of more conserved peptides (GIP, insulin, IGF1 and IGF2); and a group of highly conserved, or close to "mammalian-constant" peptides (somatostatin-14, glucagon, VIP and substance P). Hence, spread is quite extensive among the 15 peptides.

Third, even within a well defined and fully established peptide family, like the glucagon family (with glucagon, VIP and secretin, among the present examples) spread is extensive. Although strictly homologous, these hormones fall in separate sub-groups (more constant glucagon and VIP *versus* more variable secretin) and differ about 4-fold or more in variability, *i.e.*, evolutionary speed. Consequently, considerable structural differences and conservation differences are allowed in a single peptide family.

Fourth, and perhaps most important, even with its variability, C-peptide does show some conserved sequence patterns. One such pattern concerns single residues along the molecule, where five non-consecutive positions stand out. If they are to form a receptor binding site, that site is either non-continuous, or, if continuous, intervening variable residues must co-vary with corresponding variability in the receptor(s). Neither of these patterns is typical for ligand-receptor interactions within the hormone field.

However, binding through a few critical, single-residue hits and intervening residue variability occurs in peptide interactions with the major histocompatibility molecules (MHC of classes I and II[7,8]). Hence, if C-peptide participates in receptor interactions, the binding sites may perhaps be of the critical type like in MHC binding pockets rather than of continuous residue type like in many traditional peptide hormones. These findings have implications for the search regarding the mode of binding interactions in which C-peptide may participate with its putative receptor(s). Whether this pattern reflects complex interactions for C-peptide, perhaps also with associated cellular interactions involved, remains to be elucidated. In any event, the C-peptide pattern still deviates from random chance variations and may, in spite of its variability, give hope of finding putative C-peptide receptor(s), in which case their untraditional patterns may explain difficulties thus far at receptor definitions.

### References

[1] Wahren, J. *et al.* (2000). *Am. J. Physiol. Endocrinol. Metab.*, **278**, E759–E768.
[2] Ohtomo, Y. *et al.* (1996). *Diabetologia*, **39**, 199–205.
[3] Ohtomo, Y. *et al.* (1998). *Diabetologia*, **41**, 287–291.
[4] Rigler, R. *et al.* (1999). *Proc. Natl. Acad. Sci.*, USA, **96**, 13318–13323.
[5] Ido, Y. *et al.* (1997). *Science*, **277**, 563–566.
[6] Henriksson, H. *et al.* (2000). *Cell. Mot. Life Sci.*, **57**, 337–342.
[7] Guo, H.-C. *et al.* (1992). *Nature*, **360**, 364–366.
[8] Brown, J. H. *et al.* (1993). *Nature*, **364**, 33–39.

### Molecular Basis for the Insulinomimetic Effects of C-peptide

GEORGE GRUNBERGER, XIAOLING QIANG, ZHEN-GUO LI, SURESH T. MATHEWS, DIEGO SBRISSA, ASSIA SHISHEVA and ANDERS A. F. SIMA

*Center for Molecular Medicine and Genetics, Department of Internal Medicine, Department of Pathology, Department of Neurology, Department of Physiology, Morris Hood Jr. Comprehensive Diabetes Center, Wayne State University School of Medicine, Detroit, Michigan*

C-peptide, released by the $\beta$-cells of pancreatic islets, elicits salutary physiological responses in patients with type 1 diabetes mellitus. We show that synthetic rat C-peptide stimulates several insulin-like cellular effects in a rat skeletal muscle cell system. To elucidate the molecular mechanisms(s) underpinning such insulinomimetic actions as glycogen synthesis and amino acid uptake, we investigated effects of C-peptide on several elements of the insulin signaling pathway. In rat L6 cells, physiological concentrations of C-peptide (0.3-3 nM) activated insulin receptor tyrosine kinase, IRS-1 tyrosine phosphorylation, PI 3-kinase activity, MAPK phosphorylation, p90Rsk, and GSK3 phosphorylation. C-peptide-dependent effects were not reproduced by a scrambled C-peptide sequence. Only submaximal insulin concentrations (up to 10 nM) combined with submaximal C-peptide concentrations led to additive effects. Addition of C-peptide to the maximally stimulatory insulin level (100 nM) did not increase the effect of insulin, strengthening the conclusion that same signaling elements are used by both of these ligands. However, differences in specific segments of the signaling pathway (*e.g.*, lack of Akt activation by C-peptide and the bell-shaped dose response to C-peptide) indicate that C-peptide elicits some effects by a mechanism distinct from that of insulin. We speculate that C-peptide could modulate metabolic effects of insulin by enhancing them at low hormone concentrations and dampening them at high hormone levels.

### The Effect of C-Peptide on Vascular Tonicity

T. FORST

*Dept. Internal Medicine and Endocrinology, University Mainz, Mainz, Germany*

Numerous functional and structural microvascular disturbances have been reported for patients suffering from type 1 diabetes. Microvascular blood flow is regulated by endothelial cell function, the autonomic nervous function, rheologic properties and a couple of metabolic factors.

In patients suffering from diabetes mellitus type 1, skin microvascular blood flow is disturbed early in the cause of the disease with a maldistribution of nutritive and subpapillary blood flow. We investigated the influence of C-peptide administration on skin blood flow in type 1 diabetic patients and healthy control subjects by the use of laserdopplerfluxmetry and intravital capillary microscopy. In type 1 diabetic patients nutritive capillary blood flow was reduced by about 13% compared with the healthy control group. Replacement of human C-peptide completely restored nutritive capillary blood flow in these type 1 diabetic patients, while no effect was seen in the healthy control group. A linear relationship was found between plasma C-peptide levels and the capillary blood flow velocity ($r = 0.401$; $p < 0.0001$). No impact of C-peptide could be observed on subpapillary blood flow measured by laserdopplerfluxmetry. In addition, spectral analysis of flow motion revealed no significant influence of C-Peptide on neurovascular control.

There is some evidence that C-peptide action in microvascular blood flow is mediated by endothelial release of NO (see abstract of T. Kunt). Therefore, we measured plasma cGMP-levels, as a parameter of endothelial NO-secretion in type 1 diabetic patients following C-peptide administration. There was found 23% increase in plasma cGMP levels after substituation of human C-peptide infusion in type 1 diabetic patients confirming the assumption of a NO mediated vascular effect of C-Peptide.

$Na^+K^+$ATPase is involved in the regulation of microvascular blood flow and is well known to be reduced in diabetes mellitus. In our investigations, C-Peptide supplementation in type 1 diabetic patients has shown to increase erythrocyte $Na^+K^+$ATPase activity by about 100%. There was found a linear relationship between plasma C-peptide levels and erythrocyte $Na^+K^+$ATPase activity ($r = 0.46$; $p < 0.01$). Therefore, restoration of $Na^+K^+$ATPase activity may further affect microvascular blood flow by influencing vascular tonicity or erythrocyte flexibility and blood rheology.

In conclusion, C-Peptide affects microvascular blood flow by different mechanisms leading to an increase in nutritive capillary blood flow relatively to thermoregulatory or arteriovenous shunt flow. These findings might explain the beneficial effects of C-Peptide supplementation on microvascular complications in diabetes mellitus type 1.

### C-peptide and Blood Flow Regulation in Type 1 Diabetes

KARIN EKBERG

*Dept. of Surgical Sciences, Section of Clinical Physiology, Karolinska Institute, Stockholm, Sweden*

One of the early findings of C-peptide's effect in type 1 diabetes was its ability to reduce the glomerular filtration rate, increase renal blood flow and reduce the renal filtration fraction.[1] It was hypothesized that C-peptide may cause a reduction in capillary leakage, possibly by an increase in capillary recruitment. It had been described previously that diabetic patients have an increased vascular permeability.[2] To examine C-peptide's possible effect on vascular permeability, IDDM patients were studied during rhythmic forearm exercise.[3] It was found that both forearm blood flow and capillary diffusion capacity (CDC) increased during C-peptide replacement (+ 27 ± 4%, $p < 0.001$ and 52 ± 9%, $p < 0.01$, respectively) and vascular resistance decreased to levels similar to those found in healthy control subjects. The technique used did not allow a conclusion as to whether CDC was increased due to altered capillary recruitment and/or permeability. It was shown in a subsequent study using the perfused rat hindquarter model that C-peptide has no effect on vascular permeability but stimulates capillary recruitment.[4]

C-peptide exerts a stimulatory effect on forearm blood flow also in the resting state. In a recent study in Stockholm the concentration-dependent effect of C-peptide on forearm blood flow was examined in 12 IDDM patients in a placebo-controlled, double-blind study using venous occlusion plethysmography, a technique for the measurement of total blood flow in a forearm segment. It could be demonstrated that a low dose of C-peptide (1 pmol/kg/min) resulting in a plasma C-peptide level of ~0.2 nM significantly increased forearm blood flow with 15% above basal. When the C-peptide concentration was increased to ~1 nM by a higher infusion rate (5 pmol/kg/min, the dose previously used in all of the above studies) blood flow was increased by 25%. However, with an even higher dose (25 pmol/kg/min) resulting in a C-peptide concentration of ~7 nM, a significantly higher blood flow was not found. This was in accordance with previous findings by Johansson and Pernow 1999[5] where basal forearm blood flow was stimulated by C-peptide (5 pmol/kg/min) in IDDM patients (+25 ± 6%, $p < 0.01$). In that study it was also confirmed that increased blood flow was associated with a decreased vascular resistance (−17 ± 5%, $p < 0.01$).

To examine C-peptide's effect on endothelial function of the larger arteries in type 1 diabetes, Johansson and colleagues[6] determined blood flow velocity and brachial artery diameter responses following reactive hyperemia (RH) and administration of glyceryl trinitrate (GTN) using high-resolution ultrasound technology. They found that the shear stress induced arterial dilatation following RH was impaired in the diabetes patients (diabetics 2% *vs.* controls 9%), whereas the response to GTN was normal. Following C-peptide administration the basal blood flow velocity in the brachial artery was increased by 26%, $p < 0.05$, but the responses to RH and GTN were unaltered. This suggests that C-peptide acts primarily on resistance arterial vessels.

As to the mechanism for C-peptide action on blood flow, it has been demonstrated in bovine aortic endothelial cells that C-peptide stimulates on the endothelial nitric oxide synthase (eNOS) activity, resulting in an increased NO production.[7] The C-peptide induced NO release was abolished by addition of a NOS inhibitor. Recently, this was also tested in humans; intra-arterial administration of C-peptide to type 1 diabetes patients resulted in an immediate blood flow response (~30%) and when C-peptide was co-administered with L-NMMA (a NOS inhibitor) the stimulatory effect of C-peptide was abolished.[8]

In conclusion, the available data indicate that in type 1 diabetes C-peptide stimulates eNOS and release of NO primarily at the level of the resistance vessels, resulting in increased blood flow, decreased vascular resistance and increased capillary recruitment. C-peptide's effect is concentration-dependent in the range of 0-1 nM. The stimulatory effect of C-peptide may be beneficial in the prevention of diabetic complications.

### *References*

[1] Johansson, B.-L. *et al.* (1992). *Diabetologia*, **35**, 121–128.
[2] Tran-Jensen, J. (1971). *Acta Diabet. Lat.*, **8**, 192–202.
[3] Johansson, B.-L. *et al.* (1992). *Diabetologia*, **35**, 1151–1158.
[4] Lindström, K. *et al.* (1996). *Acta Physiol, Scand.*, **156**, 19–25.
[5] Johansson, B.-L. *et al.*(1999). *Acta Physiol. Scand.*, **165**, 39–44.
[6] Fernqvist-Forbes, E. *et al.* (2000). *Diabetologia* (submitted).
[7] Kunt, T. *et al.* (1998). *Diabetologia.*, **41**, A176.
[8] Johansson, B.-L. *et al.* (1999). *Diabetologia.*, **42**(suppl 1): A324.

### The Pathogenetic Role of C-peptide Deficiency in Type 1 Diabetic Neuropathy

ANDERS A. F. SIMA

*Wayne State University, Detroit, MI, USA*

C-peptide has an insulin-like effect and ameliorates the acute nerve conduction defect (NCD) in experimental and human type 1 diabetic neuropathy (DN). In this study diabetic BB/Wor-rats were treated with rat C-peptide (75 ng/kg) from onset of diabetes for 8 months (prevention-group (PG)). In a separate experiment 5 mo untreated diabetic BB/Wor-rats were started on the same C-peptide treatment continued to 8 mo of diabetes (intervention group (IG)). In the PG the NCD was significantly decreased ($p < 0.001$) compared to untreated BB/Wor-rats and was similar to that of normo-C-peptidemic and isohyperglycemic type 2 BBZ rats. This effect was associated with significant preventions of nodal changes ($p < 0.001$) including axo-glial dysjunction ($p < 0.001$), which was not different from non-diabetic control rats. Axonal atrophy and Wallerian degeneration were significantly prevented (both $p < 0.05$). In the IG the NCD decreased significantly ($p < 0.01$) during the 3 mo treatment period. Associated with the functional improvement, nodal changes improved significantly ($p < 0.001$) as did axonal degenerative changes ($p < 0.01$). C-peptide treatment in the IG resulted in a significant increase in the frequency of regenerating fibers ($p < 0.001$) compared with untreated 5 mo diabetic rats. These studies demonstrate that C-peptide replacement in type 1 diabetes significantly prevents the chronic NCD and structural changes. Furthermore, C-peptide treatment significantly improves already established chronic functional and structural abnormalities in DN. This is the first demonstration of a therapeutic improvement of established neuropathy in experimental diabetes. We conclude that C-peptide deficiency in type 1 diabetes is an important pathogenetic component in DN and that its replacement may provide a valuable adjunct to intensive insulin treatment.

### Neuroprotective Effect of C-peptide: *In Vivo* and *In Vitro* Studies

ZHEN-GUO LI, WEIXIAN ZHANG, GEORGE GRUNBERGER and ANDERS A. F. SIMA

*Center for Molecular Medicine and Genetics, Morris Hood Jr. Diabetes Center, Dept. of Pathology, Internal Medicine, Neurology, Wayne State University, Detroit Michigan*

It is known that IGF plays an important neuroprotective effect in both *in vivo* mid *in vitro* studies. We have previously shown that the IGF system is perturbed in CNS of type-I spontaneously diabetic BB/W-rats, and is associated with programmed cell death in hippocampus mid frontal cortex. In this study, we provide evidence showing that C-peptide plays a neuroprotective role by preventing the perturbed IGF system in the CNS of BB/W-rats. By using Northern blot hybridization, IGF-I, II, IGF-I receptor (IGF-IR) and insulin receptor (IR) expressions were found to be decreased in CNS of diabetic BB/W-rats compared to the age-matched control animals. In diabetic rats supplemented with 75 ng/kg/day C-peptide from onset of diabetes, the impaired mRNA expression of IGF-I, II, IGF-IR and IR was partially prevented and hippocampal neuronal apoptosis was prevented as assayed by LM-PCR assay and TUNEL staining. To elucidate the mechanism(s) underlying C-peptide's action, we examined signaling pathway of C-peptide in human neuroblastoma SH-SY5Y cells. Under 300 mM glucose, SH-SY5Y cells undergo apoptosis as shown with TUNEL/DAPI stainings. When C-peptide (10 nM) was added to the high glucose medium, the proportion of apoptotic cells are reduced compared with those without C-peptide treatment. C-peptide, in the presence of insulin, substantially increased p38 phosphorylation and significantly decreased JNK phosphorylation, indicating a bi-directional regulation of these two stress-induced MAP kinases involved in the IGF-IR mediated anti-apoptotic pathway. These data therefore suggest that the anti-apoptotic effect of C-peptide may be mediated by enhancing the activity of IGF-IR/IR signaling pathway.

### Effects of C-peptide in Experimental and Clinical Nephropathy

BO-LENNART JOHANSSON

*Section of Clinical Physiology, Department of Surgical Sciences, Karolinska Hospital, SE 171 76 Stockholm, Sweden*

An elevated glomerular filtration rate (GFR) and glomerular hypertrophy are frequent findings in the early stage of type 1 diabetic nephropathy. Insulin therapy decreases glomerular hyperfiltration but not to normal levels of GFR. C-peptide levels are low or unmeasurable in type 1 diabetes and this peptide is primarily catabolized by the kidneys. These considerations prompted us to examine the possible influence of C-peptide on renal function in streptocotozin (STZ) diabetic rats.

Short-term infusion (140 min) of C-peptide in STZ-treated animals was found to diminish GFR, reduce renal protein leakage and augment the renal functional reserve.[1] Subsequent studies involving C-peptide infusion for 14 days in rats with experimental diabetes not only confirmed the results with regard to reduction in GFR and diminished albumin excretion but also demonstrated markedly reduced renal structural changes, as evidenced by reduced glomerular hypertrophy.[2]

The above results in diabetic animals have been borne out in studies involving patients with type 1 diabetes and mild diabetic nephropathy. Thus, *i.v.*, infusion of human C-peptide (2 h) was found to be accompanied by a significant reduction of GFR (7%) and a small rise in renal blood flow (3%).[3] During more prolonged C-peptide administration (4 weeks) in type 1 patients (n = 9), provided by subcutaneous continuous rate infusion, it could be shown that both GFR and urinary albumin excretion decreased significantly, indicating a beneficial effect on glomerular permeability in type 1 diabetes.[4] However, in this study glycemic control tended to improve in the C-peptide treated group but not in the controls. This raised the question of whether the observed improvement in renal function represented a direct C-peptide effect or if it was a secondary effect related to the amelioration of blood glucose control. Therefore, a larger study was undertaken involving 22 type 1 diabetic patients with microalbuminuria receiving human C-peptide in doses calculated to restore the physiological concentration for 3 months in a double-blind randomized cross-over design. In this study glycemic control was not different during C-peptide and placebo treatment, respectively. The results showed a statistically significant, substantial reduction in microalbuminuria (40%) during C-peptide administration and no significant change during placebo treatment.[5]

The mechanism underlying the diminished albumin excretion during C-peptide administration is not known. It is possible, however, that C-peptide may exert a direct effect on the glomerular handling of albumin, as suggested from the studies of renal function in streptozotocin induced diabetic rats. A blunted $Na^+$, $K^+$-ATPase activity has been shown in animals and humans with diabetic mellitus, especially in renal and nerve tissue. It has been found that C-peptide exerts a concentration dependent stimulatory effect on $Na^+$, $K^+$-ATPase activity in rat renal tubular cells. Similar findings have also been obtained in glomeruli from streptozoto-cin-induced diabetic rats. Finally, evidence is now available, indicating that C-peptide may stimulate endothelial nitric oxide synthase.[6] In this context it is of interest that specific binding of C-peptide to membrane-bound receptors has been demonstrated in the nanomolar concentration range for renal cells.[7] Thus, it may be hypothesized that C-peptide has the potential to influence membrane permeability and transport as well as stimulate regional blood flow, possibly resulting in improvements in renal function in the diabetic state.

In conclusion, the combined animal experiment and human trial data demonstrate that C-peptide replacement in type 1 diabetes and mild nephropathy exerts a beneficial effect on renal structure and function. The possibility should be considered that C-peptide should be provided to type 1 diabetes patients to prevent or retard the development of diabetic nephropathy.

### *References*

[1] Sjöquist, M. *et al.* (1998). *Kidney International*, **54**, 758–764.
[2] Johansson, B.-L. *et al.* (2000). *Diabetologia*, **43**, Suppl. 1, A37.
[3] Johansson, B.-L. *et al.* (1992). *Diabetologia*, **35**, 121–128.
[4] Johansson, B.-L. *et al.* (1993). *J. of Clinical Endocrin. and Metab.*, **77**, 976–981.
[5] Johansson, B.-L. *et al.* (2000). *Diabetic Med.*, **17**, 181–189.
[6] Kunt, T. *et al.* (1998). *Diabetologia*, **41**, A176.
[7] Rigler, R. *et al.* (1999). *Proc. Natl. Acad. Sci.*, USA, **96**, 1318–1323.

### Grb10, a Potential Partner of P85 P13-Kinase in Insulin-Stimulated Metabolism

SUJOY BHATTACHARYA, YOUPING DENG, RAMA SWAMY ORRE, CHONG BAL and HOLMO RIEDEL

*Department of Biological Sciences, Wayne State University, Detroit, MI*

Growth factor receptor binding protein 10 (Grb10) has been implicated as a signaling mediator in association with several mitogenic peptide hormone receptors including the insulin receptor. In response to insulin Grb10 is specifically recruited into a complex with the insulin receptor. However, a role of Grb10 in the regulation of insulin-stimulated metabolic responses has not been reported. In this study we have investigated the putative role of one Grb10 variant in insulin-induced metabolism with several independent experimental approaches.

We found that over-expression of complete Grb10 cDNA in Rat 1 fibroblasts over-expressing insulin receptors or in Rat L6 myocytes stimulated insulin-induced cellular responses including glycogen, synthesis, glucose uptake, amino acid uptake and lipogenesis consistent with a role of Grb10 in insulin-induced metabolism. In contrast, putatively dominant-negative cell membrane-permeable Grb10 SH2 and Pro-rich domain fusion peptides substantially inhibit insulin-stimulated glycogen synthesis which indicated a role of these domains in insulin-regulated metabolism, consistent with a stimulatory role of complete Grb10. Association of complete Grb10 with the regulatory subunit p85 of P13-kinase was observed in response to insulin stimulation. Both the Grb10 SH2 and Pro-rich regions inhibited the association between Grbr10 and p85. Consistent with their implicated roles in Grb10-p85 association, both the Grb10 SH2 and Pro-rich regions substantially inhibited insulin-stimulated P13-kinase activity, suggesting functional roles in P13-kinase activation. The observed association between Grb10 and p85 may be indirect and involve other signaling adapters. One candidate is Gabl which may link Grb10 to the MAP kinase pathway. In this context both the Grb10 SH2 domain and the Pro-rich region were found to inhibit MAP kinase activation by insulin. Over-expression of Grb10 in PC12 neural cells stably expressing an inactive p85 P13-kinase restored insulin-induced glycogen synthesis. A possible interaction of the Grb10 Pro-rich region with the SH3 domain of p85 could contribute to P13-kinase activation and the transduction of the insulin-induced metabolic signal. When combined, our findings implicate Grb10 as a potential partner of p85 P13-kinase with a role in signaling mechanisms that mediate insulin-stimulated metabolic responses.

### C-peptide Inhibits Protein Tyrosine Phosphatase and Promotes Glycogen Synthesis

ZHEN-GUO LI, XIAOLING QIANG, SURESH MATHEWS,
ANDERS A. F. SIMA and GEORGE GRUNBERGER

*Center for Molecular Medicine and Genetics, Morris Hood Jr. Comprehensive Diabetes Center, Depts. of Internal Medicine, Pathology, Neurology, Wayne State University, Detroit, Michigan*

In spite of extensive investigation, the mechanism(s) mediating C-peptide's beneficial effects on diabetic neuropathy and nephropathy is (are) not clear. We have demonstrated previously that C-peptide exerts insulino-mimetic effects, such as activation of glycogen synthesis and amino acid uptake. Direct evidence that C-peptide binds to the insulin receptor, however, has not been established. In this study, we demonstrate that C-peptide inhibits protein tyrosine phosphatase (PTP) activity in L6 myoblasts, in a dose-related manner. The maximal inhibitory effect was shown in ranges of 0.3-3.0 nM equivalent to its physiological concentrations. As a result of the PTP inhibition, insulin receptor autophosp-horylation and IRS-1 phosphorylation were enhanced. Consistent with these findings, C-peptide at the above concentrations enhanced glycogen synthesis and potentiated the effect of insulin (10 nM) in L6 myoblasts. These results indicate that C-peptide may either act via the insulin receptor directly or cross talk with an activated cognate C-peptide receptor at a proximal level of the insulin-signaling pathway.

### Molecular Pathology of the Node of Ranvier in Type I Diabetic Neuropathy: A Role for Insulin and C-peptide Deficiencies?

CHRISTOPHER R. PIERSON[a], WEIXIAN ZHANG[a], YUICHI MURAKAWA[a],
JOHN WAHREN[b] and ANDERS A. F. SIMA[a]

[a]*Wayne State University, Department of Pathology and Morris Hood Jr. Comprehensive Diabetes Center, Detroit, MI;*
[b]*Department of Surgical Sciences, Karolinska Hospital, Stockholm, Sweden*

C-peptide replacement in type I BB/W-rats prevents both the initial metabolic and the chronic structural changes at the node of Ranvier. Nodal integrity depends on specialized molecules including ankyrinG and caspr. AnkyrinG interacts with $Na^+$ channels, $Na^+K^+$-ATPase, cell adhesion and axonal cytoskeletal molecules, sequestering them to the node of Ranvier. Caspr localizes to the septate-like junctions that form between axons and myelin loops. These interactions are responsible for the regional specializations at the node and permit the timely propagation of action potentials. The insulin receptor is localized to paranodal cell membranes[1] and C-peptide potentiates the insulin signaling pathway,[2] suggesting that insulin signaling may be critical to nodal molecule activity. *In vitro* studies have suggested a potential interaction between caspr and PI3K via SH3 domains.[3] We hypothesize that the activities of these nodal molecules are altered in type I neuropathy and that insulin and/or C-peptide deficiencies may underlie these changes. Immunoblotting (sciatic nerve) and semi-quantitative RT-PCR (DRG) reveal no difference in the expression levels of ankyrinG; and its associated molecules ($Na^+$ channels, $Na^+K^+$-ATPase, L1, and NCAM) between control; C-peptide treated (75 ng/kg/d), and untreated diabetic BB/W-rats at 2 and 8 mo. of diabetes. AnkyrinG is post-translationally modified at serine residues by O-linked N-acetylglucosamine (O-G1cNAc). Immunoprecipitation of ankyrin[G] in SH-SY5Y cells treated with C-peptide (3.3 nM), insulin (4.0 nM), or both for 2 hr showed an increase in O-G1cNAc modification of ankyrin[G], with insulin alone. Whereas cells exposed for 20 hr showed decreased O-G1cNAc modification by insulin alone, which was enhanced when C-peptide was added. Therefore, C-peptide may potentiate the effects of insulin long term (20 hr) but blunt insulin's effects short term (2 hr). Immunoblotting (sciatic nerve) and semi-quantitative RT-PCR (DRG) revealed no difference in the expression level of caspr at 2 mo. however, at 8 mo. caspr protein expression was decreased in diabetics. Immunohisto-chemical studies show that caspr is laterally displaced from the paranode in diabetic rats at 2 mo., an alteration that is partially rectified by C-peptide. These findings suggest that localization and post-translational modifications may be more important than alterations in the expression of these nodal molecules in type I diabetic neuropathy.

### References

[1] Sugimoto, Y. *et al.* (2000). *Diabetes Metab Rev.*, **16**, 354–363.
[2] Sima, A. A. F. *et al.* (1998). *Diabetologia*, **41**[Suppl. 11], Al17.
[3] Peles, E. *et al.* (1997). *EMBO*, **16**, 978–988.

### C-peptide Signaling–is it Mediated Through Insulin Receptor?

XIAOLING QIANG, SURESH T. MATHEWS, ZHEN-GUO LI, ANDERS A. F. SIMA and GEORGE GRUNBERGER

*Center for Molecular Medicine and Genetics, Department of Internal Medicine, Department of Pathology, Wayne State University School of Medicine Detroit, Michigan 48201*

Earlier we have shown that synthetic rat C-peptide stimulates several insulin-like cellular effects *viz.*, glycogen synthesis and amino acid uptake, in a rat L6 skeletal muscle cells. Binding of insulin to its receptor results in activation of insulin receptor tyrosine kinase (IR-TK), followed by phosphorylation of intracellular substrates, propagating receptor signals throughout the cell. Our goal was to verify if C-peptide activated proximal steps of the insulin signal transduction pathway. We demonstrate that physiological concentrations of C-peptide (0.3-3 nM) activated IR autophosphorylation, TK, and IRS-1 tyrosine phosphorylation in a dose-dependent manner in rat L6 cells, indicating that C-peptide mimics insulin's effects of IR activation. To understand the mechanism of C-peptide-induced IR activation, binding studies were performed. In L6 cells, unlabeled C-peptide displaced bound [$^{125}$I] C-peptide in a whole cell competitive binding assay, suggesting the existence of C-peptide receptors. Further, physiological concentrations of unlabeled C-peptide displaced [$^{125}$I]-insulin, bound to wheat germ agglutinin-purified IR. Could our results suggest that C-peptide's insulinomimetic effects are mediated via binding of C-peptide to IR?

### IGF-I Inhibits High Glucose Induced Oxidative Stress and Mitochondrial Dysfunction in a Model of Diabetic Neuropathy

J. W. RUSSELL, D. GOLOVOY, P. MAHENDRU and E. L. FELDMAN

*Department of Neurology, University of Michigan, Ann Arbor, MI 48109*

In streptozotocin diabetic rats hyperglycemia is associated with apoptotic changes in dorsal root ganglion neurons such as DNA fragmentation and disruption of mitochondria (Mt). These events are associated with loss of sensory neurons and clinical neuropathy. We find that hyperglycemic induced Mt dysfunction is a critical precursor for neuronal apoptosis. Using confocal microscopy of cultured dorsal root ganglion neurons exposed to 20 mM glucose in serum/insulin free defined medium, there is a rise in $H_2DCFDA$ levels (a measure of reactive oxygen species [ROS]), that peaks at 6 h, followed by a decline in ROS corresponding to an increase in neuronal apoptosis over the following 72 h. The rise in ROS is coupled with a five fold increase in Mt size corresponding to Mt swelling and release of cytochrome-c into the neuronal cytosol, events that are p I recursors to apoptosis. The rise in ROS and swelling of Mt is inhibited by 3 μM myxothiazole, which blocks Mt superoxide generation, as well as by 50–100 μM bongkrekic acid (BKA) which blocks the ANT/VDAC complex of the Mt and prevents depolarization of the Mt membrane, Insulin-like growth factor-I (IGF-I) mimics the anti-oxidant properties of myxothiazole and the Mt membrane stabilizing effect of BKA. IGF-I inhibits Mt enlargement and depolarization ($p < 0.01$), and cytochrome-c release from the Mt. These results indicate that an early glucose induced rise in ROS is associated with Mt swelling *in vitro*, and can be blocked by anti-oxidants and IGF-I. These findings support the growing evidence of increased oxidative stress in diabetic neuropathy and indicate that JGF-I can stabilize neuronal Mt and reduce oxidative processes leading to neuronal dysfunction. This work was supported by NIH NS01938, VA Merit and Juvenile Diabetes Foundation International (JWR); NIH NS32843, Juvenile Diabetes Foundation International and the American Diabetes Association (ELF).

### IGF-I Inhibits Programmed Cell Death Associated with High Glucose Induced Oxidative Stress

J. W. RUSSELL, D. GOLOVOY, A. R. BERENT and E. L. FELDMAN

*Department of Neurology, University of Michigan, Ann Arbor, MI 48109*

In previous studies, we have shown that hyperglycemia induces mitochondrial (Mt) enlargement and disruption in streptozotocin treated diabetic rats. Furthermore, these changes are associated with histological evidence of DNA fragmentation and cell death. The role of hyperglycemia in inducing Mt dysfunction and apoptosis was further characterized in vitro using dorsal root ganglion (DRG) cultures. In the presence of 20 mM added glucose there is a 40% increase in caspase-3 cleavage measured using the CM I antibody (gift from Dr. A. Srinivasan, IDUN Pharm, La Jolla, CA) at 6 h and 55% at 24 h ($p < 0.001$). Myxothiazole (3 μM), an inhibitor of Mt oxidation, blocks release of cytochrome-c from the intermitochondrial space into the cytosol, and inhibits cleavage of caspase-3 up to 24 h. Furthermore, 50–100 μM bongkrekic acid (BKA) which blocks the ANT/VDAC complex of the Mt and prevents depolarization of the Mt membrane, inhibits caspase-3 cleavage up to 24 h ($p < 0.001$). Apoptosis induced by 20 mM added glucose, as measured by the percent of TUNEL positive neurons, is blocked by addition of the pan caspase inhibitor Boc-Asp-FMK and IGF-I. Thus *in vitro*, PCD associated with glucose induced oxidative stress or Mt depolarization, can be blocked by anti-oxidants, Mt stabilizing agents and IGF-I. These findings support the association between oxidative stress and PCD in models of diabetic neuropathy. This work was supported by NIH NS01938, VA Merit and Juvenile Diabetes Foundation International (JWR); NIH NS32843, Juvenile Diabetes Foundation International and the American Diabetes Association (ELF).

### Specific Regulation by Ras of Cytokine-Mediated Nitric Oxide Generation in the Pancreatic *β* Cell

MARIE TANNOUS[a], MICHAEL POPOFF[b] and ANJAN KOWLURU[a]

[a]*Department of Pharmaceutical Sciences, Wayne State University and John D. Dingell VA Medical Center, Detroit, MI 48201. USA;* [b]*Unit of Microbial Toxins, Pasteur Institute, 25 Rue Docteur Roux, 75724 Paris, Cedex 15, France*

Insulin-dependent diabetes mellitus develops as a consequence of the selective destruction of insulin producing *β* cells due to autoimmune aggression. It has been shown that such cytotoxic effects are mediated by cytokines [*e.g.*, interleukin-1*β*] secreted by the infiltrating immune cells. Cytokine-induced islet *β* cell death is attributed primarily to the induction of nitric oxide synthase [*iNOS*] leading to the generation of nitric oxide [NO] within the cell, which in turn, culminates in cell death by apoptosis and necrosis. In the present study, we have shown that exposure of insulin secreting *β* [HIT-T15] cells to interleukin-1*β* [IL-1*β*] results in a time- and dose-dependent increase in NO release. Such effects by IL-1*β* on NO release were mediated by induction of iNOS from these cells. Preincubation of HIT-T15 cells with *Clostridium sordelli* lethal toxin- 82, which irreversibly glucosylates and inactivates low molecular weight GTP-binding proteins, such as Ras, Rap, and Rac, but not Cdc42, completely abolished IL-1*β*-induced NO release from these cells. Pre-exposure of HIT-T15 cells to *Clostridium sordelli* lethal toxin-9048, which monoglucosylates and inhibits Ras, Cdc42, Rac, but not Rap also attenuated IL-1*β*-mediated NO release. These data indicate that activation of Ras and/or Rac may be necessary for IL-1*β*-mediated NO release. Further, two structurally dissimilar inhibitors of Ras function, namely manumycin A and damnacanthal inhibited, in a concentration-dependent manner, the IL-1*β* -mediated NO release from these cells. Together, our data provide evidence, for the first time, that Ras activation is an obligatory step in IL-1*β* -mediated NO release [presumably] subsequent dysfunction of the pancreatic *β* cell. It is likely that activation of other G-proteins [*e.g.*, Rac] is downstream to the site of regulation by Ras. These studies provide basis for future investigations to understand the mechanism of cytokine-induced *β* cell death leading to the onset of insulin-dependent diabetes mellitus.

### Hoechst 33342 Induces Apoptosis and Alters Insulin and Nitric Oxide Release in Syrian Hamster Bets Cells

XINBO ZHANG[a], ALISON LONG[a], J. DOUGLAS FERRY[a], VIKTOR BROVKOVYCH[b], TADEUSZ MALINSKI[b] and FREDERICK L. KIECHLE[a]

[a]*Department of Clinical Pathology, William Beaumont Hospital, Royal Oak M1;*
[b]*Center for Biomedical Research, Oakland University, Rochester, MI*

Hoechst 33342 (H342), but not Hoechst 33258 (H258), induces apoptosis and inhibits topoisomerase I in a variety of cell lines. To investigate the effect of H342 and H258 on Syrian hamster beta cells (HIT-T15), cell viability was detected by trypan blue exclusion and DNA fragmentation by 1.5% agarose gel electrophoresis. After H342 or H258 treatment, insulin release (measured in media) and intracellular insulin levels (measured in cell lysate) were determined by a solid phase, competitive immunoassay (Coat-A-Count®, Diagnostic Products Corp). Nitric oxide release was measured using an electrochemical porphyrinic microsensor. Cell death and DNA fragmentation into multiples of 180-200 base pairs were both increased in a time-dependent manner after treatment with a lethal dose (26.7 μmol/L H342) for 6-24 hours when compared with the control group, H258-treated cells or sublethal dose (4.5/μmol/L) of H342. Insulin release was increased by 0.5 fold after treatment with the lethal dose of H342 for 1 hour and then decreased by 1.8 fold after 6 hours when compared with the control group, sublethal dose or H258 group (4.5 or 26.7 μmol/L). However, both intracellular and total insulin levels measured in the cell lysates of Syrian hamster beta cells were significantly decreased in the presence of the lethal dose of H342 for 1 hour and 3 hours, respectively. H258 did not alter the insulin concentration compared to the control. Maximal increase in nitric oxide (NO) release was 120% (4.5 μmol/L H342) at 3 hours; 89% (26.7 μmol/L H342) at 2 hours. H258 did not alter NO release. These findings are consistent with the activation of proteases which degrade intracellular proteins (insulin) and genomic DNA by protease-activated nuclease during H342-induced apoptosis. The relationship between NO production and insulin degradation requires further investigation.

### The Effect of C-peptide on Nitric Oxide

THOMAS KUNT

*Dept. of Endocrinology, University of Mainz, Mainz, Germany, Institute for Clinical Research and Development, Mainz, Germany, Hospital of Annweiler, Germany*

There is increasing evidence for biological functions of human C-peptide. Recently, we have described that proinsulin C-peptide increases nutritive capillary blood flow in type I diabetes patients, whereas it has no such effect in non-diabetic subjects. The aim of the presented studies was to elucidate cellular mechanisms of this vasodilator effect *in vitro* by measuring the nitric oxide (NO) mediated increase of cGMP production in a RFL-6 reporter cell assay, by demonstrating endothelial calcium influx with the Fluo-3 technique and by demonstrating the effect of C-peptide on erythrocyte deformability. C-peptide increased the release of NO from bovine aortic endothelial cells (BAEC) in a concentration- and time-dependent manner. At physiological C-peptide concentrations, endothelial NO production was approximately doubled ($192 \pm 15\%$ *vs.* control; $p < 0.01$). The NO release was abolished by the inhibitor of NO synthase $N^G$-nitro-L-arginine or when $Ca^{2+}$ was removed from the medium superfusing the BAEC. C-peptide stimulated the influx of $Ca^{2+}$ into the BAEC as demonstrated by the Fluo-3 technique. C-peptide did not change NOS III mRNA levels after 1, 6 or 24 h. These data indicate that C-peptide is likely to stimulate the activity of the $Ca^{2+}$-sensitive endothelial NO synthase by increasing the influx of $Ca^{2+}$ into endothelial cells. Furthermore, we were able to show that erythrocyte deformability is attenuated in normoglycemic type I diabetic patents compared to non-diabetic control subjects. If C-peptide was added to the erythrocyte suspensions of type 1 diabetic patients, the deformability of the red blood cells increased to normal levels that were not different from those of control subjects. This effect was abolished if ouabain, an antagonist of Na/K-ATPase, was added, We suggest that these effects may contribute to the increase in skin and muscle blood flow previously demonstrated in man *in vivo* and may be induced by a stimulation of calcium influx and an activation of Na/K-ATPase and nitric oxide synthase as well.

